# Technical data on the inhibition properties of some medicinal plant extracts towards caseinolytic protease proteolytic subunit of *Plasmodium knowlesi*

**DOI:** 10.1016/j.dib.2021.107588

**Published:** 2021-11-20

**Authors:** Raimalynah Abd Razak, Meiny Suzery, Rafida Razali, Zarina Amin, Ruzaidi Azli Mohd Mokhtar, Ping Chin Lee, Cahyo Budiman

**Affiliations:** a Biotechnology Research Institute, University Malaysia Sabah, Jalan UMS, Kota Kinabalu, Sabah 88400, Malaysia; b Department of Chemistry, Faculty of Sciences and Mathematics, Diponegoro University, Jl. Prof. Soedarto, SH, Tembalang, Semarang 50275, Indonesia

**Keywords:** *Plasmodium knowlesi*, Malaria, Caseinolytic protease, Plant extracts

## Abstract

Proteolytic subunit of the caseinolytic protease system of *Plasmodium knowlesi* (Pk-ClpP; EC 3.4.21.92) is considered a viable target for antimalarial drug development to eradicate *P. knowlesi* malaria infection in Malaysia and Southeast Asian region. Inhibition of this system leads to a disruption in the protein homeostasis molecular machinery and therefore be lethal for the parasite. While plants are considered excellent sources of bioactive compounds exhibiting inhibition activity towards Pk-ClpP, many local medicinal plants remain unexplored. This article expands the data collected from the inhibition properties of the methanolic extract of *Asystasia gangetica* (Chinese Violet), *Alstonia scholaris* (Pulai Tree), *Piper retrofractum* (Javanese Long Pepper) and *Smallanthus sonchifolius* (Yacon) towards Pk-ClpP. These plants are widely found in Malaysia and Indonesia and have been traditionally used in various medical treatments. The present dataset showed that the extracts contained phenolic and flavonoid compounds in various concentrations, whereby *S. sonchifolius* was found to have the lowest content of phenolic and flavonoid contents, while *A. gangetica* and *A. scholaris* were statistically comparable, yet higher than *P. retrofactum* and *S. sonchifolus*. Further inhibition data assay towards Pk-ClpP revealed that *A. gangetica, A. scholaris* and *P. retrofactum* demonstrated remarkable inhibition activity with IC_50_ values of 39.06 ± 1.98, 48.92 ± 1.52, and 87.63 *±* 3.55, respectively. However, the inhibition activity of these extracts was significantly lower than a serine protease inhibitor of phenylmethylsulfonyl fluoridenone (PMSF). Meanwhile, *S. sonchifolus* did not exhibit significant inhibition activity towards Pk-ClpP. In addition, Pk-ClpP was not inhibited by a cysteine protease inhibitor of E64.

## Specifications Table

**Table:** 

Subject	Biological sciences
Specific subject area	Biochemistry
Type of data	Table Figure
How data were acquired	The purity of protein used in this study was visualized using Gel DocTM XR+ imager (Biorad, CA, USA). Total phenolic and flavonoid contents of the extract as well as protein concentration were measured using Lambda 35 Perkin-Elmer UV-Vis spectrophotometer (MA, USA). Data of IC_50_ was calculated using GraphPad Prism (version 9.0), GraphPad Software Inc. (CA, USA). While ANOVA statistical analysis was conducted using the Minitab 15 Statistical Software (PA, USA).
Data format	Raw Analyzed
Parameters for data collection	The parameters include: (1). Purity of the target protein (Pk-ClpP); (2). Total phenolic and flavonoid contents of the methanolic extracts from the plants; (3). Inhibition properties of the extracts, with positive and negative controls, against enzymatic activity of Pk-ClpP.
Description of data collection	The pure recombinant Pk-ClpP was obtained from heterologous expression from the other works. The purity of this protein was qualitatively checked using SDS-PAGE visualised under DocTM XR+ imager (Biorad, CA, USA). The total phenolic and flavonoid contents of the extracts were measured using the Folin– Ciocalteu and aluminium nirate colorimetric methods, respectively, and expressed as the amount (mg) of gallic acid equivalents (GAE) or quercetin equivalents (QE) per g of plant extract, respectively. The inhibition properties were measured by determining the enzymatic activity of Pk-ClpP in the absence or in the presence of various concentration of plant extracts. The inhibition properties were quantified as IC_50_ referring to the concentration of extract required to inhibit 50% of Pk-ClpP activity.
Data source location	Experiments and data collection were performed at the Integrated Laboratory, Diponegoro University, Semarang, Indonesia and Biotechnology Research Institute, Universiti Malaysia Sabah, Kota Kinabalu Malaysia.
Data accessibility	With the article

## Value of the Data

• The present data provide scientific evidence on the promising properties of *Asystasia gangetica* (Chinese Violet), *Alstonia scholaris* (Pulai Tree), *Piper retrofractum* (Javanese Long Pepper) and *Smallanthus sonchifolius* (Yacon) to be further explored and studied for the development of antimalarial drugs.
• The present data should benefit other researchers working on plant compound-based drug discovery for malaria to expand their compound sources. This data also benefits the countries suffering from *P. knowlesi* infection, yet rich in biodiversity, to harness the local medicinal plants to combat the infection.
• The present data might be used as the reference in the isolation of the bioactive compounds from the extracts that contribute to the inhibition activity. The data also might be used as the basis to study the inhibition properties of the extract against other malaria parasites.

## 1 Data Description

Flavonoid and phenolic compounds are known to be the most important groups of secondary metabolites and bioactive compounds in plants [1]. It is therefore important to determine the composition of these compounds in the extracts used in this study. Table 1 indicated that the total phenolic contents among the plant extracts were found to be significantly different (*P* < 0.01). The total phenolic and flavonoid contents of *A. gangetica* and *A. scholaris* were found to be statistically comparable, yet significantly higher than that of P. *retrofractum* and *S. sonchifolius*. Overall, the sequence for total phenolic and flavonoid contents is in the following order: *A. gangetica ∼ A. scholaris > P. retrofractum> S. sonchifolius*. Table 1 also indicated that the variation of phenolic content (4.38 – 56.17 mg/g) among the samples was higher than that of the flavonoid content (1.73–32.33 mg/g). Notable, other the specific bioactive compounds of the extracts exhibiting antimicrobial properties were also possible to present. Nevertheless, no report so far for the specific compounds with antiparasitic activity from *A. gangetica, A. scholaris, P. retrofractum* ref*,* and *S. sonchifolius.* Accordingly, in this study, measurement was limited to phenolic and flavonoid compounds as a general secondary metabolite in the extracts.

**Table 1:** Total phenolic and flavonoid contents of the extract.

Plant extract	Total phenolic content (mg GAE / g)	Total flavonoid content (mg QE / g)
*Asystasia gangetica*	56.17 ± 0.90A	32.33 ± 1.96A
*Alstonia scholaris*	50.50 ± 1.3A	34.65 ± 0.59A
*Piper retrofractum*	25.08 ± 2.22B	12.84 ± 1.68B
*Smallanthus sonchifolius*	4.38 ± 0.79C	1.73 ± 0.70C

* The values with different letters in a column are significantly different (*P* < 0.01).

**Fig. 1:**
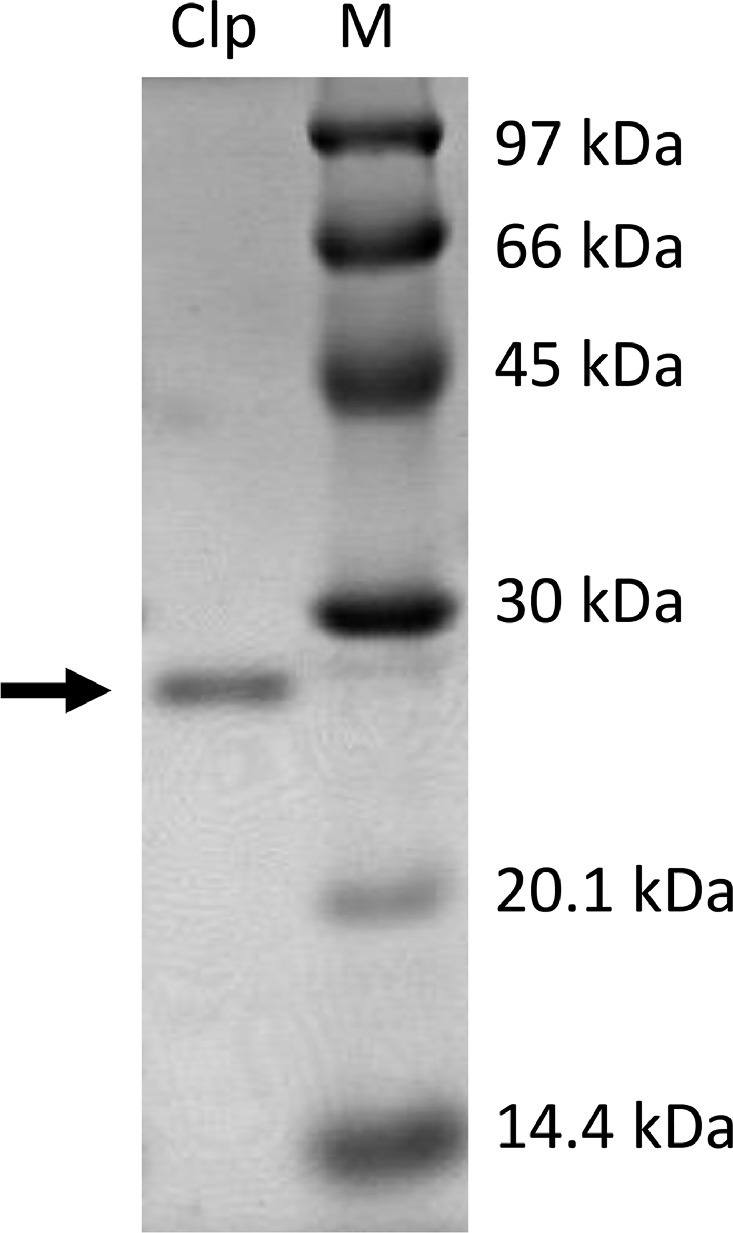
Purity of Pk-ClpP (Lane Clp) under 15% SDS-PAGE. The marker of proteins with known sizes (kDa) are shown on Lane M.

As the extracts used in this study were confirmed to contain phenolic and flavonoid compounds, *albeit* in varied concentrations, it is then interesting to confirm whether the extract exhibits inhibition activity towards Pk-ClpP. For this purpose, high purity of recombinant Pk-ClpP was used for the inhibition test (Fig. 1). As shown in Fig. 1, the purity of Pk-ClpP used in this study is considerably high as indicated by the absence of remarkable bands from other proteins.

**Fig. 2:**
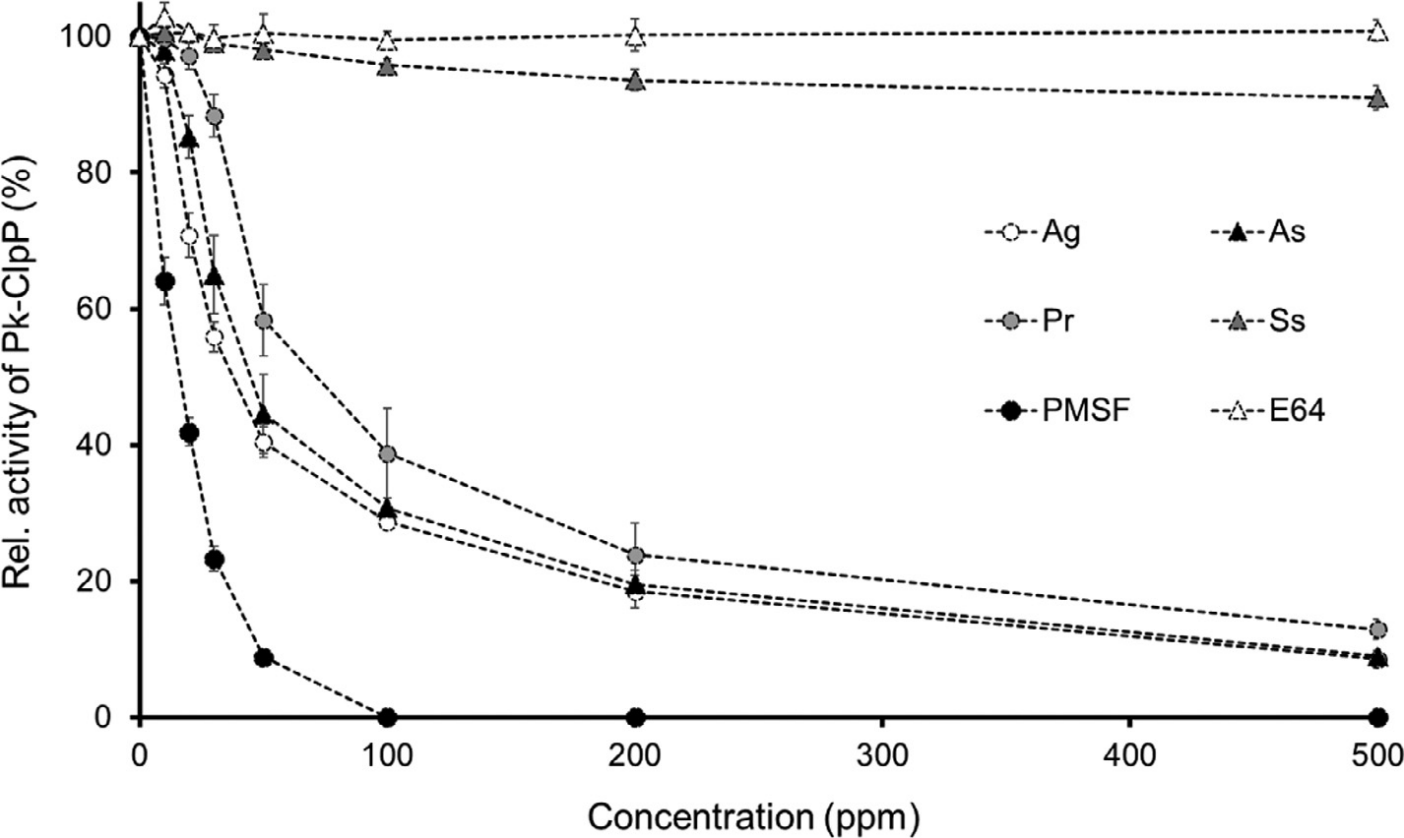
Relative activity of Pk-ClpP in the presence of various concentration of *Asystasia gangetica* (Ag), *Alstonia scholaris* (As), *Piper retrofractum* (Pr), *Smallanthus sonchifolius* (Ss), serine protease inhibitor phenylmethylsulfonyl fluoride (PMSF) and cysteine protease inhibitor (E64).

Fig. 2 showed that *P. retrofractum, A. gangetica, A. scholaris* and positive control of phenylmethylsulfonyl fluoride (PMSF; a serine protease inhibitor) demonstrated remarkable inhibition activity toward Pk-ClpP. On the other hand, inhibition *S. sonchifolius* did not inhibit Pk-ClpP well. Up to 500 ppm of *S. sonchifolious*, the residual activity of Pk-ClpP remains about 90%, which implied that the extract did not exhibit significant inhibition activity. Meanwhile, the negative control of E64 (a cysteine protease inhibitor) did not inhibit Pk-ClpP. As shown in Fig. 2, the inhibition of the extracts and PMSF toward Pk-ClpP was clearly in a concentration-dependent fashion. Accordingly, the IC_50_ value was able to be calculated for PMSF, *P. retrofractum, A. gangetica,* and *A. scholaris*, but not for *S. sonchifolius* and E64 (Table 2). Based on the IC_50_ values, the best inhibition activity was observed in *A. gangetica.* None of the extracts exhibited inhibition activity better than PMSF.

**Table 2:** IC_50_ value of the plant extracts toward Pk-ClpP.

Plant extract	IC_50_ (ppm)
*Asystasia gangetica*	39.06 ± 1.98A
*Alstonia scholaris*	48.92 ± 1.57B
*Piper retrofractum*	87.63 ± 3.55C
*Smallanthus sonchifolius*	N.D
Phenylmethylsulfonyl fluoride (PMSF)	8.96 ± 0.51
E64	N.D

N.D = IC_50_ value of S*. sonchifolius* and E64 were not feasible to be calculated.

## 2 Experimental Design, Materials and Methods

### 2.1 Plant extract preparation

All the plant extracts were methanolic extracts and obtained from the Integrated Laboratory of the University of Diponegoro, Indonesia. A methanol extract of Javanese Long Pepper *(Piper retrofractum*) was prepared from the chili fruit, obtained from Wonogiri, Central Java, Indonesia, as reported by Cahyono et al. [2]. The methanolic extract of Chinese Violet (*Asystasia gangetica*) and Yacon (*Smallanthus sonchifolius*) was prepared from the leaf part based on Suzuki et al. [3] and Mendoza et al. [4], respectively. Meanwhile, the methanolic extract of Pulai Tree (*Alstonia scholaris*) was obtained from the bark as described by Bello et al. [5]. All the extracts were evaporated to remove the solvent, followed by the freeze-dried and kept at -80 °C freezer before further usages.

### 2.2 Total phenolic compound

The extraction of total phenolic was performed using the Folin– Ciocalteu assay following the method of Siddiqui et al. [6] with some modifications. In total, 100 µl of each extract (1 mg/ml) was added to a test tube containing 50 µl of the phenol reagent (1 M). Further, 1.85 ml of distilled deionized water was added to the solution and allowed to stand for 3 min after being vortexed; then 300 µl Na_2_CO_3_ (20% in water, v/v) was added and vortexed and the final volume (4 ml) was obtained by adding 1.7 ml of distilled deionized water. A reagent blank was prepared using distilled deionized water. The final mixture was vortexed, and then incubated for 1 h in the dark at room temperature. The absorbance was measured at 725 nm using Lambda 35 Perkin-Elmer UV-Vis spectrophotometer (MA, US). A standard curve was prepared using 0, 65.5, 125, and 250 mg/l gallic acid in methanol: water (50:50, v/v). Total phenolic content is expressed as the amount of gallic acid equivalents (GAE) in mg/g of plant extract. All determinations were performed in triplicate.

### 2.3 Total flavonoid compounds

The total flavonoid content in extracts was determined using the aluminium nirate colorimetric method as described by Moreno et al. [7] with some modifications. Briefly, a 0.5 ml sample (1 mg/ml) was mixed with 0.1 ml of 10% aluminium nitrate and 0.1 ml of potassium acetate (1 M), and 4.3 ml of 80% ethanol was added to make a total volume of 5 ml. The mixture was vortexed, and the solution was allowed to stand for 40 min for the reaction at room temperature. The absorbance was measured spectrophotometrically at 415 nm. Total flavonoid content as the amount of of quercetin equivalents (QE) per gram of plant extract. A standard curve was prepared using 0, 5, 10, and 100 mg/l solutions of quercetin. All determinations were performed in triplicate.

### 2.4 Protein preparation

The protein used in this study was purified recombinant full-length of Pk-ClpP (Gene ID: OTN65960.1) provided by Angelesa Runin (Biotechnology Research Institute, Universiti Malaysia Sabah). This protein was expressed using *Escherichia coli* BL21(DE3) and purified using Ni^2+^-affinity chromatography according to El Bakkouri et al. [8]. The protein was dissolved in 20 mM phosphate buffer pH 8.0. Prior to the analysis, the purity of the protein was checked using a 15% gel of SDS-PAGE. To remove aggregated proteins and other particles, the sample was centrifuged at 30,000 g for 30 min at 4°C followed by filtration of the supernatant using 0.22 µm filters (Millipore Corp., Bedford, MA).

### 2.5 Quantitative assay of protein

The total protein content of the samples was determined by Lowry's method [9]. The protein standard used was Bovine Serum Albumin (BSA).

### 2.6 Preparation of casein agar plates

The stock of casein was prepared by dissolving alkaline soluble casein in distilled deionized water. The insoluble portion was dissolved by the addition of the alkali. The pH was adjusted to 8.0 with 0.1 M NaOH [10]. The casein was then added into 2% of bacto agar to have the final concentration of 3%, followed by sterilization at 121 °C for 20 min. After the sterilization, the mixture was cooled down to about 60 °C followed by the addition of kanamycin antibiotic at 35 mg/mL of final concentration and then poured into plates and allowed to harden. The wells (4 mm in diameter) on the plate were then made using a sterile cork borer.

### 2.7 Well-diffusion assay

Pure Pk-ClpP (0.5 mg/mL at a final concentration) was firstly mixed with various concentrations of plant extract, ranging from 10 to 500 ppm. The mixture was then incubated at 37 °C for 15 min and then deposited at the well of casein agar plates. The plates were then incubated at 37 °C and observed for two days for the halo zone formation. The activity was denoted as unit activity (U), in which 1 U is defined as the amount of enzyme needed to produce 1 mm of halo zone in 1 h that indicates hydrolysis of casein. The activity of Pk-Clp in the absence of any plant extract (control) was adjusted as 100% of activity. The relative activity of Pk-Clp in the presence of plant extract was calculated as the ratio to the activity of the control. As a positive control, a serine protease inhibitor of PMSF (Sigma-Aldrich, MA, USA) was used in this study. Meanwhile, a cysteine protease inhibitor of E64 (Sigma-Aldrich, MA, USA) was used as a negative control. The IC_50_ value, which refers to the concentration of the sample required to reduce 50% of the catalytic activity [11,12], was calculated using a four-parameter logistic curve under the GraphPad Prism (version 9.0), GraphPad Software Inc. (CA, USA). The assay was done in triplicate.

### 2.8 Data analysis

Data obtained were presented as mean ± standard deviation. The experiment was performed under an experimental design of a completely randomized design [13]. Significance differences among the values were determined using a one-way analysis of variance (ANOVA). Duncan's multiple range tests were used to assess the significant differences with the Minitab 15 Statistical Software (PA, USA).

## Ethics Statement

This article does not contain any studies with human or animal subjects performed by the any of the authors.

## Supplementary Materials

The authors confirm that the raw data of this study are available as supplementary materials.

## CRediT Author Statement

**Raimalynah Abd Razak:** Investigation, Formal analysis, Writing – original draft; **Meiny Suzery:** Visualization, Writing – review & editing; **Rafida Razali:** Visualization, Formal analysis; **Zarina Amin:** Data curation, Writing – review & editing; **Ruzaidi Azli bin Mohd Mokhtar:** Supervision, Conceptualization, Data curation, Writing – review & editing, Funding acquisition; **Ping Chin Lee:** Supervision, Conceptualization, Data curation, Funding acquisition, Writing – review & editing; **Cahyo Budiman:** Conceptualization, Methodology, Formal analysis, Data curation, Supervision, Writing – review & editing, Funding acquisition, Writing – original draft.

## Declaration of Competing Interest

The authors declare that there is no conflict of interest.

## Appendix Supplementary materials

Image, application 1
