# Supplementary material for: Technical data on the inhibition properties of some medicinal plant extracts towards caseinolytic protease proteolytic subunit of Plasmodium knowlesi
Source: Data Brief. 2021 Nov 20;39:107588. doi: 10.1016/j.dib.2021.107588 (PMC8627994; doi:10.1016/j.dib.2021.107588)
Supplement: Supplementary file 1 [file mmc1.docx]

**Supplementary Material**

**Data sheet data on the inhibition properties of some medicinal plant extracts towards caseinolytic protease proteolytic subunit of *Plasmodium knowlesi***

**Figure S1.** Raw figure of purified Pk-ClpP under 15% SDS-PAGE


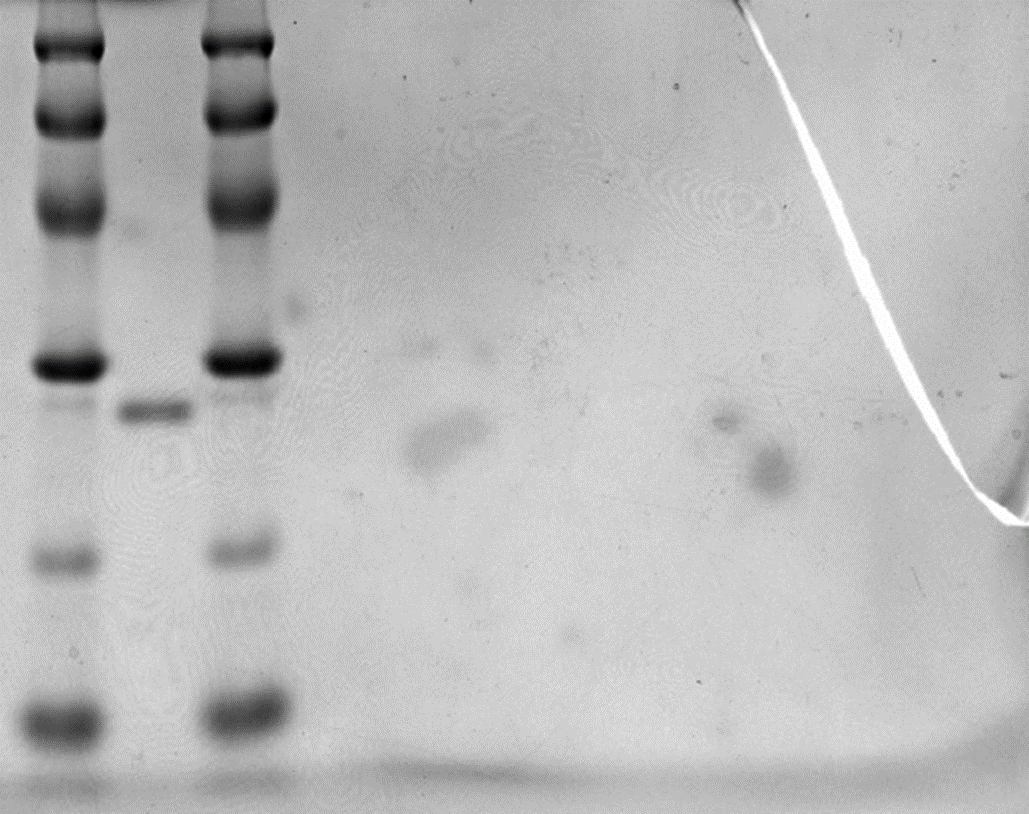


**Table S1.** Raw data of phenolic content of the extracts

| Plant extract | Total phenolic content (mg GAE/g) | | | | |
| --- | --- | --- | --- | --- | --- |
|  | Rep1 | Rep2 | Rep3 | Average | Std. Dev |
| *Asystasia gangetica* | 57.11 | 56.09 | 55.31 | 56.17 | 0.90266 |
| *Alstonia scholaris* | 49.32 | 50.3 | 51.9 | 50.5067 | 1.30236 |
| *Piper retrofractum* | 27.31 | 22.86 | 25.07 | 25.08 | 2.22502 |
| *Smallanthus sonchifolius* | 4.87 | 3.47 | 4.81 | 4.38333 | 0.79154 |

*Rep = replication

**Table S2.** Raw data of flavonoid content of the extracts

| Plant extract | Total flavonoid content (mg QE/g) | | | | |
| --- | --- | --- | --- | --- | --- |
|  | Rep1 | Rep2 | Rep3 | Average | Std. Dev |
| *Asystasia gangetica* | 33.81 | 30.11 | 33.06 | 32.3267 | 1.95597 |
| *Alstonia scholaris* | 35.21 | 34.7 | 34.04 | 34.65 | 0.5866 |
| *Piper retrofractum* | 10.9 | 13.9 | 13.72 | 12.84 | 1.6825 |
| *Smallanthus sonchifolius* | 2.37 | 0.98 | 1.86 | 1.73667 | 0.70316 |

*Rep = replication

**Table S3.** Raw data of relative activity of Pk-ClpP in the presence of various concentration of *Asystasia gangetica* and *Alstonia scholaris* extracts

| Concentration (ppm) | *Asystasia gangetica* | | | | | *Alstonia scholaris* | | | | |
| --- | --- | --- | --- | --- | --- | --- | --- | --- | --- | --- |
|  | Rel. activity (%) | | | | | Rel. activity (%) | | | | |
|  | Rep 1 | Rep 2 | Rep 3 | Average | Std-Dev | Rep 1 | Rep 2 | Rep 3 | Average | Std-Dev |
| 0 | 100.00 | 100.00 | 100.00 | 100.00 | 0.00 | 100.00 | 100.00 | 100.00 | 100.00 | 0.00 |
| 10 | 96.31 | 93.09 | 93.41 | 94.27 | 1.77 | 98.09 | 98.31 | 97.03 | 97.81 | 0.68 |
| 20 | 72.31 | 73.11 | 67.08 | 70.83 | 3.28 | 88.72 | 82.68 | 84.47 | 85.29 | 3.10 |
| 30 | 53.64 | 58.09 | 55.83 | 55.85 | 2.23 | 70.84 | 64.89 | 59.37 | 65.03 | 5.74 |
| 50 | 39.01 | 43.06 | 39.21 | 40.43 | 2.28 | 51.09 | 40.26 | 42.31 | 44.55 | 5.75 |
| 100 | 28.90 | 28.02 | 29.08 | 28.67 | 0.57 | 32.16 | 30.71 | 29.30 | 30.72 | 1.43 |
| 200 | 18.01 | 21.07 | 16.31 | 18.46 | 2.41 | 20.55 | 20.83 | 17.31 | 19.56 | 1.96 |
| 500 | 9.50 | 9.07 | 7.04 | 8.54 | 1.31 | 9.31 | 8.32 | 9.37 | 9.00 | 0.59 |

**Table S4.** Raw data of relative activity of Pk-ClpP in the presence of various concentration of *Piper retrofractum* and *Smallanthus sonchifolius* extracts

| Concentration (ppm) | *Piper retrofractum* | | | | | | *Smallanthus sonchifolius* | | | | | |
| --- | --- | --- | --- | --- | --- | --- | --- | --- | --- | --- | --- | --- |
|  | Rel. activity (%) | | | | | | Rel. activity (%) | | | | | |
|  | Rep 1 | Rep 2 | Rep 3 | Average | Std-Dev | Rep 1 | | Rep 2 | Rep 3 | Average | Std-Dev |  |
| 0 | 100.00 | 100.00 | 100.00 | 100.00 | 0.00 | 100.00 | | 100.00 | 100.00 | 100.00 | 0.00 |  |
| 10 | 100.08 | 99.32 | 99.57 | 99.66 | 0.39 | 101.31 | | 99.89 | 100.31 | 100.50 | 0.73 |  |
| 20 | 98.10 | 94.90 | 98.31 | 97.10 | 1.91 | 100.09 | | 100.45 | 101.09 | 100.54 | 0.51 |  |
| 30 | 91.31 | 88.53 | 85.08 | 88.31 | 3.12 | 99.32 | | 99.30 | 98.35 | 98.99 | 0.55 |  |
| 50 | 60.31 | 62.31 | 52.39 | 58.34 | 5.25 | 97.49 | | 98.30 | 98.40 | 98.06 | 0.50 |  |
| 100 | 34.22 | 35.80 | 46.30 | 38.77 | 6.57 | 94.95 | | 95.71 | 96.71 | 95.79 | 0.88 |  |
| 200 | 25.61 | 27.46 | 18.49 | 23.85 | 4.74 | 92.41 | | 93.09 | 95.31 | 93.60 | 1.52 |  |
| 500 | 14.30 | 11.36 | 13.09 | 12.92 | 1.48 | 89.05 | | 91.61 | 92.31 | 90.99 | 1.72 |  |

**Table S5.** Raw data of relative activity of Pk-ClpP in the presence of various concentration of phenylmethylsulfonyl fluoride (PMSF) and E64

| Concentration (ppm) | Phenylmethylsulfonyl Fluoride (PMSF) | | | | | E64 | | | | | |  |
| --- | --- | --- | --- | --- | --- | --- | --- | --- | --- | --- | --- | --- |
|  | Rel. activity (%) | | | | | Rel. activity (%) | | | | | |  |
|  | Rep 1 | Rep 2 | Rep 3 | Average | Std-Dev | | Rep 1 | Rep 2 | Rep 3 | Average | Std-Dev | |
| 0 | 100.00 | 100.00 | 100.00 | 100.00 | 0.00 | | 100.00 | 100.00 | 100.00 | 100.00 | 0.00 | |
| 10 | 62.31 | 68.05 | 61.82 | 64.06 | 3.46 | | 102.01 | 101.31 | 105.32 | 102.88 | 2.14 | |
| 20 | 40.72 | 44.31 | 40.68 | 41.90 | 2.08 | | 101.08 | 100.98 | 99.83 | 100.63 | 0.69 | |
| 30 | 25.31 | 21.68 | 22.94 | 23.31 | 1.84 | | 98.03 | 101.86 | 99.25 | 99.71 | 1.96 | |
| 50 | 7.81 | 9.03 | 9.62 | 8.82 | 0.92 | | 102.67 | 97.31 | 101.32 | 100.43 | 2.79 | |
| 100 | 0.00 | 0.00 | 0.00 | 0.00 | 0.00 | | 98.32 | 99.08 | 100.91 | 99.44 | 1.33 | |
| 200 | 0.00 | 0.00 | 0.00 | 0.00 | 0.00 | | 99.94 | 102.61 | 98.03 | 100.19 | 2.30 | |
| 500 | 0.00 | 0.00 | 0.00 | 0.00 | 0.00 | | 102.60 | 100.01 | 99.85 | 100.82 | 1.54 | |

**Table S6.** Raw data of IC_50_ of the extracts against Pk-ClpP

| Plant extract | Total flavonoid content (mg QE/g) | | | | |
| --- | --- | --- | --- | --- | --- |
|  | Rep1 | Rep2 | Rep3 | Average | Std. Dev |
| *Asystasia gangetica* | 37.57 | 41.31 | 38.3 | 39.06 | 1.98 |
| *Alstonia scholaris* | 50.42 | 49.04 | 47.29 | 48.92 | 1.57 |
| *Piper retrofractum* | 85.71 | 91.72 | 85.45 | 87.63 | 3.55 |
| *Smallanthus sonchifolius* | ND | ND | ND | ND | ND |
| Phenylmethylsulfonyl fluoride (PMSF) | 8.83 | 9.53 | 8.53 | 8.96 | 0.51 |
| E64 | ND | ND | ND | ND | ND |

*Rep = replication; ND= not determined
